# Topical Administration of Cannabidiol: Influence of Vehicle-Related Aspects on Skin Permeation Process

**DOI:** 10.3390/ph13110337

**Published:** 2020-10-23

**Authors:** Antonella Casiraghi, Umberto M. Musazzi, Giorgio Centin, Silvia Franzè, Paola Minghetti

**Affiliations:** Department of Pharmaceutical Sciences, Università degli Studi di Milano, via Mangiagalli 25, 20133 Milan, Italy; umberto.musazzi@unimi.it (U.M.M.); giorgio.centin@studenti.unimi.it (G.C.); silvia.franze@unimi.it (S.F.); paola.minghetti@unimi.it (P.M.)

**Keywords:** cannabidiol, topical administration, in vitro permeation, human skin, solutions, semisolid dosage forms, (trans)dermal patch, Hildebrand solubility parameter

## Abstract

Cannabidiol (CBD) is a non-psychoactive cannabinoid isolated from Cannabis sativa which, given its claimed beneficial properties and therapeutic potential, has lately aroused considerable attention from the scientific community. Starting from the little literature evidence, the main purpose of this study was to investigate the topical administration of CBD, with particular focus on the influence of vehicle-related aspects on the skin permeation process. This could provide useful information for the design of suitable drug delivery systems which could be used in developing topical medicines and cosmetics. In vitro human skin permeation studies were conducted using modified Franz diffusion cells to compare the performance of four solutions and two semisolid formulations. The Hildebrand solubility parameter was used to better understand the thermodynamic aspects implied in the partitioning process of the cannabinoid compound into the skin. It was interestingly found that a hydrophilic gel, mostly consisting of propylene glycol (79%, *w*/*w*), can be an optimal choice for the topical administration of CBD. Moreover, the feasibility of the preparation of CBD-loaded (trans)dermal patches, made with new printing technology, was also demonstrated.

## 1. Introduction

Cannabidiol (CBD), a non-psychoactive cannabinoid isolated from the Cannabis sativa plant, has attracted great attention due to several beneficial effects reported in the literature.

CBD has been investigated for the treatment of several neuropsychiatric disorders, and non-communicable diseases [1,2,3]. Several studies on different animal models and some clinical trials have been conducted [1,2,3]. In 2019, the European Medicines Agency (EMA) approved the first CBD-based orphan medicine, Epidyolex^®^ (GW Pharma International B.V., Amersfoort, the Netherlands): an oral solution (100 mg/mL) indicated for use as adjunctive therapy of seizures associated with Lennox-Gastaut and Dravet syndromes [4]. Starting from that, several oral formulations have been developed for therapeutic purposes [2]. However, low bioavailability of CBD (13–19%) was observed after gastrointestinal intake due to its very-limited water solubility in gastrointestinal fluids and the important first-pass metabolism [5].

On the contrary, the (trans)dermal administration of CBD is advantageous in terms of pharmacokinetics in comparison to the gastroenteric one [2]. Indeed, significant plasma concentrations of CBD were observed in animal models after the transdermal gel application [6,7,8]. Moreover, starting from the discovery of novel biological and pharmacological properties, topical CBD has been proposed for several skin-related therapeutic indications [2,3]. Based on its ability to modulate the skin inflammatory response, CBD efficacy in the treatment of several dermatological conditions, including psoriasis, atopic dermatitis, and acne, has been investigated in vivo [2,3,9]. In an early clinical study on 20 volunteers suffering from various skin diseases, Palmieri et al. observed that a three-month treatment with a CBD ointment improved patients’ clinical outcomes and quality of life by providing an anti-proliferative and anti-inflammatory action [10]. Moreover, the discovery that CBD acts synergistically with bacitracin against *Staphylococcus aureus* and other Gram-positive bacteria [11] opened novel frontiers in the treatments of cutaneous infections, benefiting from both its antimicrobial and anti-inflammatory activity [12].

Taking into account the scientific and commercial interest in possible CBD topical applications, several liquids (e.g., oil, spray) and semisolid formulations (e.g., creams, gels, patches) have been developed and patented [2]. Many of them have been developed to be marketed as medicinal products [2,3] or as cosmetics. Indeed, several skincare products have also been placed on the market, relying on CBD claimed anti-seborrheic, anti-aging, antioxidant, and skin-protective properties [13,14].

Despite this, a limited number of in vitro and in vivo studies aimed to investigate the impact of the vehicle on the CBD permeation profile through models of human skin have been available in the literature [15]. In order to reach the therapeutic target, a molecule has to permeate through the stratum corneum (SC) and distribute through the underlying layers of the epidermis. In this context, the vehicle significantly influences the partitioning process of a molecule based on its physicochemical characteristics [16].

This study aimed to investigate the influence of vehicle-related aspects on CBD percutaneous absorption. Four different solvent systems and two semisolid formulations, namely an ointment and a hydrophilic gel, were compared in terms of drug permeation and retention in the human epidermis. Taking into consideration the potential systemic use of CBD by transdermal administration, a (trans)dermal patch was also prepared by an innovative printing technique [17]. Particular attention was given to formulation aspects related to solubility and thermodynamic activity of the permeant. For this purpose, the Hildebrand solubility parameter [18] was determined for each placebo vehicle to estimate the thermodynamic activity of the cannabinoid compound.

## 2. Results

Table 1 summarizes the results of solubility and skin permeation tests. Four different solvent systems were considered, two of lipophilic nature: liquid paraffin (S_1_), virgin olive oil (S_2_); and two of hydrophilic nature, namely propylene glycol 80% (S_3_), polyethylene glycol (PEG) 400 80% (S_4_). Among the above, S_1_ and S_3_ showed a significantly better performance than the other two solutions in terms of CBD permeation rate (*p* < 0.005), and thus they can be considered the most appropriate solvent systems for cutaneous administration of CBD. While the performance of S_1_ and S_3_ was similar in terms of steady-state flux (J) and the cumulative permeated amount at 24 h (Q_p,24_), the propylene glycol solution provided a significantly higher retained amount at 24 h (Q_r,24_) of CBD (*p* < 0.001). It is also noteworthy that S_1_ and S_3_ were the vehicles in which CBD was less soluble.

Starting from the two best performing vehicle solutions, namely liquid paraffin, and propylene glycol/water mixture, two semisolid preparations were proposed: a lipophilic ointment (F_1_) and a hydrophilic gel (F_2_). The composition of each preparation is reported in Table 2. F_1_ was prepared following a monograph of Italian Pharmacopoeia [19], increasing liquid paraffin content as much as needed to solubilize the entire CBD strength and thus ensure proper incorporation in the preparation. F_2_ was obtained by simply dispersing a gelling agent in the S_3_ mixture; this same semisolid formulation was used in a previous in vivo animal study performed by Paudel et al. [7].

The hydrophilic gel proved to be the best performing composition against the lipophilic ointment and the (trans)dermal patch (F_3_) in terms of CBD skin retention (*p* < 0.005) and permeation rate (*p* < 0.001). Semisolid formulations exhibited a lower overall performance when compared to the corresponding solutions. Transition from liquid paraffin to lipophilic ointment led to a significant reduction of both CBD retained amount (*p* < 0.05) and permeation rate (*p* < 0.001). A performance drop was also observed consequent to the transition from aqueous solution to hydrophilic gel in terms of CBD skin retention (*p* < 0.05); a slight lag time increase was observed too (*p* < 0.05). As mentioned before, F_2_ outperformed the other two formulations, while F_1_ and F_3_ gave comparable results in terms of CBD skin retention. Indeed, a significant difference between F_1_ and F_3_ was only found when comparing permeation flux (*p* < 0.001), with F_1_ having the highest *J* value. The lag time differences among the three vehicles (F_1_, F_2_, F_3_) were not statistically significant.

The overall results showed that the permeation/retention profile of CBD could be modulated by changing the formulation composition. In this light, the Q_r,24_/J ratio is a simple parameter for choosing the most appropriate formulation based on the localization of the therapeutic target [17]. If the delivery system must ensure high skin retention in the upper layers of the skin, formulations with a Q_r,24_/J >> 1 should be preferred. Otherwise, the lower the Q_r,24_/J, the higher the promotion of the drug permeation through the lower epidermal layers. As reported in Table 1, all tested formulation prevalently sustained CBD retention into the human epidermis. In the case of lipophilic formulations, the Q_r,24_/J ratio increased after the transition from liquid to semisolid (e.g., S_1_ vs. F_1_). An opposite trend was observed for hydrophilic formulations (e.g., S_3_ vs. F_2_). The patch formulation resulted in the highest Q_r,24_/J ratio.

## 3. Discussion

Several systems have already been discussed in the literature or are available on the market for the cutaneous administration of CBD [2,3]. However, no systematic or mechanistic studies have been reported concerning the impact of formulation composition on the CBD permeation and retention patterns. Such lack of information may also have an impact on the rationalization of the CBD strength in the delivery systems: for example, in semisolid preparations tested in clinical trials, the CBD ranged from 1% to 10% of the formulation weight [6,20,21].

Defining the composition of a suitable vehicle for topical administration of a newly found active compound, such as CBD, requires finding a balance between various elements, including solubility and thermodynamic activity of the drug as well as penetration enhancer content and occlusive effect of the vehicle. Active compound solubility in the vehicle should be adequate to contain the full intended drug dose in solute form while ensuring the physical stability of the preparation at the same time. The composition of the four solutions was defined based on these requirements; in fact, preliminary solubility tests (data not reported) were previously led considering different solvents and concentrations. These data also allowed us to define the composition of the receiving phase for permeation experiments.

Thermodynamic activity is the driving force of the drug partitioning process into the SC; as a matter of fact, the interfacial transport of a drug from a vehicle to the SC is promoted when thermodynamic activity increases, providing either a higher accumulation into the skin and/or a higher permeation through the skin. Indicatively, the thermodynamic activity of a solute gets higher when concentration is increased, but it is also strongly affected by the physicochemical properties of the vehicle [22].

A way to consider the relationship between molecule structure and thermodynamic activity is the solubility parameter (δ), which is a simply calculable value that could be useful in skin permeation studies. Drug partition between vehicle and SC has been related to the difference between δ values of the active ingredient and the placebo formulation [23]. In addition to δ values of CBD and excipients, Table 3 shows the solubility parameter of O-acylglucosylceramide (CER), one of the predominant components of the intercellular lipids in the SC. For this reason, the δ value of CER was used as a reference for skin lipids; this is mainly relevant in the case of highly lipophilic compounds such as CBD, as the intercellular route could be considered the preferential path through the skin [24]. Firstly, it can be observed that CBD and CER having similar δ values (Δδ_CBD- skin_ = 1.13 cal/cm^3^) indicates that the cannabinoid has a great affinity for the SC, this feature makes the molecule itself a promising drug for cutaneous administration but not for transdermal absorption, as partition into the deeper and more hydrophilic layers of the epidermis may be less favorable. Indeed, there is a close relation between solubility parameter and o/w partition coefficient; as a matter of fact, δ value can be considered as an indicator of the lipophilicity of a compound: the lower the δ value, the higher the affinity for lipophilic substances [25].

In addition to solubility data and skin permeation parameters of CBD, Table 1 also reports δ values of the placebo correspondents of each tested solution and vehicle formulation as well as Δδ^2^ values, which were calculated as reported in Section 4.9.

The Δδ^2^ value can also be regarded as an index of the affinity between the solute and solvent system. This consideration is consistent with what was observed in the solubility tests, as a greater difference corresponded to a lower solubility. Moreover, while the concentration of CBD is the same, it is possible to estimate the following order of thermodynamic activity for the permeant in each solution. Equation (1):

S_4_ ≤ S_2_ < S_1_ < S_3_ (1)

as a larger difference in δ values corresponds to a higher thermodynamic activity of CBD in the vehicle. This agreed with what was observed in permeation tests, as liquid paraffin (S_1_) and propylene glycol 80% (S_3_) proved to be the best performing vehicle solutions, providing higher CBD skin retention and permeation rate as against the other two solvent systems.

A good correlation was found between the retained amount of CBD and the square of the difference between δ values of the cannabinoid and placebo solutions (Δδ^2^) Equation (2):

(2) $$Q_{r,24}=1.247\;\left(\Delta \delta^{2}\right)-0.707\;\left(n=4;\;F=41.42;\;p=0.023;{\;r}^{2}=0.954\right)$$

This suggests that a high thermodynamic activity is mainly relevant to promote the accumulation of CBD in the SC and the viable epidermis (SCE), as already observed with other compounds [30]. On the other hand, no correlation was found between Δδ^2^ and J (nor Q_p,24_) values. The lack of correlation is due to the same permeation performance of liquid paraffin and propylene glycol solutions, suggesting that other aspects play a significant role in CBD permeation through the skin, for example, excipients that also act as penetration enhancers and/or occlusion. Indeed, some chemicals (such as propylene glycol) can penetrate the SC, while others (including liquid paraffin) can limit perspiration leading to an increase in SC water content. In both cases the action of these compounds may result in a structural alteration of the lipid matrix, temporarily influencing permeability to some active compounds. The effects of both solvents on the skin are reported in the literature. It is known that propylene glycol can promote percutaneous absorption of some types of molecules, as permeation of the solvent through the SC could alter the thermodynamic activity of the active compound in the skin which would in turn modify the driving force of diffusion [31]. Moreover, Hoelgaard et al. observed that propylene glycol can diffuse through the full thickness of SCE in a short period, therefore, the solvent can act as sort of a carrier for the active substances dissolved in it [32]. Penetration enhancement induced by liquid paraffin is mainly due to its occlusive properties. As noted by Patzelt et al., the lipophilic mixture is not able to penetrate beyond the first upper layers of the SC [33], therefore it is possible to speculate that the influence on skin permeability resulting from a possible interaction between alkanes composing liquid paraffin and lipids of the extracellular matrix might be very limited.

Permeation of CBD was also investigated by using three vehicles which should simplify the administration of the cannabinoid. In the case of lipophilic ointment (F_1_) and hydrophilic gel (F_2_), CBD was solubilized in the same liquid excipients which were previously tested as solutions, whereas it was molecularly dispersed in the adhesive matrix in the case of the patch (F_3_). Focusing the attention on semisolid preparations, we can observe that δ values explained the observed differences again. Indeed, the greater the Δδ^2^ values, the higher the permeated and retained amounts of CBD. These data related to thermodynamic activity were found to be consistent with what was then observed in the in vitro tests. Retained and permeated CBD amounts determined by the hydrophilic gel were respectively more than two and three times higher than those determined by the ointment. It is noteworthy that the trend of Q_r,24_ values of semisolid formulations was consistent with that observed for the corresponding solutions (F_1_ vs. S_1_; F_2_ vs. S_3_). The difference in permeation performance between solution and semisolid formulation can be due to various contributing factors. As well as with the active ingredient, all excipient contained in the system matrix can potentially interact with the SC, influencing the partitioning process of the drug itself.

On the other hand, the (trans)dermal patch (F_3_) resulted in worse performance both in terms of permeation and retention than semisolid preparations. Such an outcome may be due to the vehicle determining a lower thermodynamic activity of the permeant, as suggested by Δδ^2^ values. However, due to the different physical state of the formulation matrixes (F_1_ and F_2_ vs. F_3_), such a hypothesis should be verified by further studies. Regardless, it is worth noting that the applied dose of the patch was lower if compared to that of the other formulations. Indeed, although CBD concentration in the matrixes was the same (1%, *w*/*w*), the applied dose of the semisolid formulations was 10 times greater than that of the (trans)dermal patch. Despite unfavorable dosing, the (trans)dermal patch exhibited a slightly better performance in terms of permeation rate if compared to that of virgin olive oil (S_2_) or PEG 400 solution (S_4_), which seemed to grant more favorable thermodynamic conditions (*p* > 0.05). It was then supposed that tributyl citrate may act as a mild penetration enhancer towards CBD through a carrier mechanism like that described for propylene glycol. Indeed, the traces of such plasticizer were found in the receiver phase of in vitro permeation studies, suggesting that it diffused through the SCE as well.

Evidence in the literature highlighted that CBD was quite unstable in a solution for a long period [34,35]. Even if an in-depth investigation of the chemical stability of CBD loaded in the tested formulation has not been performed yet, preliminary data highlighted that CBD was stable over both the preparation process and the permeation experiments (total impurities < 2% of CBD assay). Such findings were particularly relevant in the case of printed patches, suggesting that the exposition of CBD to high temperature for a limited period did not significantly affect the impurity pattern of molecules. Further studies are needed to determine the long-term stability of the tested formulations.

## 4. Materials and Methods

### 4.1. Materials

CBD was kindly gifted by Indena Spa (Milan, Italy). Cetostearyl alcohol, cetomacrogol 1000, and white petrolatum were purchased from A.C.E.F. Spa (Fiorenzuola d’Arda, Italy). Eudragit RL 100 was purchased from Evonik Industries AG (Essen, Germany). Polyethylene glycol 400 was purchased from Caesar & Loretz GmbH (Hilden, Germany). Propylene glycol and liquid paraffin were purchased from Carlo Erba Reagents Srl (Milan, Italy). Medium-viscosity hydroxyethyl cellulose was purchased from Farmalabor Srl (Canosa di Puglia, Italy). Virgin olive oil was purchased from Sergio Fontana Srl (Canosa di Puglia, Italy). Tributyl citrate was purchased from Vertellus Specialties Ltd. (Workington, UK). High-performance liquid chromatography (HPLC)-grade acetonitrile and 96% ethanol were purchased from VWR International Srl (Milan, Italy). Methanol was purchased from Merck Life Science Srl (Milan, Italy). Silicon polyester release liner was purchased from IBSA Spa (Lodi, Italy). CoTran™ polyethylene backing film 9720 was purchased from 3M Company (Saint Paul, USA). All other chemicals were of analytical grade and were used without further purification.

### 4.2. CBD Solubility at Saturation

The equilibrium solubility of CBD in each of the above-mentioned solvent systems was determined by the shake-flask method. An excess quantity of the cannabinoid compound was added to 1 mL of each test solution in a glass vial. The sample solution was capped and left under magnetic stirring at room temperature for 24 h. In the case of aqueous solutions, the supernatant fluid was then filtered (0.45 μm PP syringe filter) and properly diluted in methanol before being analyzed by HPLC. In the case of oily solutions, the supernatant fluid was filtered (0.45 μm PVDF syringe filter); then, 60 μL of the filtered solution was withdrawn and added to 5 mL of methanol; the mixture was vortexed for 1 min and allowed to stand for 1 min three times, then it was centrifuged (4000 rpm, 2 min); the supernatant fluid was sampled and properly diluted in methanol before being analyzed by HPLC. Each solubility value was calculated as the mean of three independent shake-flask experiments, considering any dilution factors and an extraction efficiency factor in case of oily solutions.

### 4.3. Preparation of Solutions

CBD solutions (1%, *w*/*w*) were prepared using different solvent systems: liquid paraffin (S_1_), virgin olive oil (S_2_), propylene glycol/purified water (80/20, *v*/*v*) (S_3_), PEG 400/purified water (80/20, *v*/*v*) (S_4_). The composition of each solution is summarized in Table 2. The solutions were obtained by dissolving a weighted drug amount in each solvent media by magnetic stirring overnight.

### 4.4. Preparation of Semisolid Formulations

CBD was incorporated at a concentration of 1% (*w*/*w*) into a hydrophobic ointment and a hydrophilic gel (Table 2). The hydrophobic ointment (F_1_) was prepared by dissolving CBD in liquid paraffin, adding the other components, and heating the mixture under magnetic stirring until completely melted; the system was then cooled to room temperature while stirring constantly with a spatula. The hydrophilic gel (F_2_) was prepared by dissolving CBD in a propylene glycol/purified water solution (80/20, *v*/*v*), hydroxyethylcellulose was then slowly added; the mixture was vigorously shaken, slightly heated, and sonicated until homogeneous in appearance.

### 4.5. Preparation of (Trans)Dermal Patch

CBD was incorporated at a concentration of 1% (*w*/*w*) into a pressure-sensitive adhesive (PSA) matrix to obtain a patch, which was prepared by the novel technique named hot-melt ram extrusion printing [17]. The PSA matrix was obtained by mixing CBD and Eudragit RL in a mortar by geometric trituration, then adding tributyl citrate and continuing to stir. Relative quantities of each component are reported in Table 2 (F_3_). Once a uniform dough was obtained, the mixture was transferred to the extrusion chamber, which was thermostatically controlled at 100 °C. The melted material was then extruded through a 1.8 cm length 19 G needle at a rate of 50 mm^3^/s and deposited on a polyethylene backing layer. The ram speed and the chamber temperature were controlled by Repetier-Host 2.0.1 software (Hotword GmbH, Germany); the matrix dimension (5 cm × 2.5 cm × 50 μm), and the number per each print were designed by using 3D builder v18 (Microsoft Inc, USA). Finally, printed patches were paired with a silicon polyester release liner, cut, and sealed with an aluminum packaging foil.

### 4.6. In Vitro Skin Permeation Study

The in vitro permeation and retention studies were performed under occlusive conditions by using modified Franz diffusion cells (permeation area: 0.636 cm^2^; receptor chamber volume: 3 mL) and the human epidermis as a membrane.

Human skin originated from the abdominal region of a donor who underwent cosmetic surgery. Full-thickness skin was sealed in evacuated plastic bags and stored at −20 °C within 6 h after removal. SCE samples were prepared following an internal standard procedure [36]. Briefly, before the experiments, the skin was thawed at room temperature and excess fat was carefully removed. The SCE sections were cut into squares of about 4.0 cm^2^ and, after immersion in 60 °C water for 1 min, the epidermis was gently separated from the remaining tissue with forceps.

At the beginning of the permeation experiment, tested formulations were applied onto the SC of each epidermis sample. In particular, solution aliquot (0.2 mL) was loaded directly in the donor compartment of each cell, whereas about 200 mg of semisolid composition was applied by using an excavated polyethylene disc (1 mm thick) as a frame, while 18 mm wide discs were cut from printed (trans)dermal patches and applied after removing the release liner. Receptor compartments were filled with degassed purified water/ethanol solution (50/50, *v*/*v*), in which the solubility of CBD was experimentally determined as 2.24 ± 0.08 mg/mL. Special care was taken to avoid the formation of air bubbles between the membrane and the solution in the receptor compartment. The gap between upper and lower parts of the Franz cells was sealed with Teflon tape and Parafilm^®^ (VWR International Srl, Milan, Italy), the two parts were then fastened together using a clamp. The system was kept at 37 °C by a circulating water bath so that the membrane surface temperature resulted to be 32 ± 1 °C throughout the experiment. At predetermined times (1, 3, 5, 7, and 24 h), 200 μL samples were withdrawn from the receiver compartment and analyzed by HPLC. The withdrawn aliquots were replaced with the same volume of fresh receiver medium. Sink conditions were maintained throughout the experiments.

The cumulative amount of CBD permeated through the skin per unit area (Q_P_) was calculated from drug concentration in the receiving medium and plotted as a function of time. The steady-state flux (J) was determined as the slope of the linear portion of the plot. Lag time was determined as the x-intercept of the slope at a steady state. The obtained results were expressed as an average of parallel experiments performed at least in triplicate.

### 4.7. In Vitro Skin Retention Study

At the end of permeation experiments, the epidermis sheet was removed from the Franz diffusion cell and each side was gently treated with 5 mL of methanol to wash out the unabsorbed drug. Subsequently, the membrane was dried, thinly sliced, and placed in 4 mL of fresh methanol. The suspension was soaked in a sonicator for 1 h and then maintained at 2–8 °C for 24 h. Finally, the supernatant was filtered (0.45 μm PP syringe filter) and analyzed by HPLC. The retained CBD amount (Q_r,24_) was expressed as micrograms per unit area of SCE. Results were expressed as an average of parallel experiments performed at least in triplicate.

### 4.8. HPLC Analysis

HPLC analysis was run using an HP 1100 ChemStation system (Agilent Technologies Inc, USA), acetonitrile/phosphate buffer pH 3.0 (75/25, *v*/*v*) was used as mobile phase at a flow rate of 1.5 mL/min, injection volume was set at 20 μL. The UV detector was set at a wavelength of 215 nm. A reverse-phase C8 column (InertClone™, 5 μm, 150 × 4.6 mm; Phenomenex Inc, Torrance, USA) was used. The retention time of the CBD was 4.0 min. Two calibration curves were made in the overall range of 0.02–100 μg/mL.

### 4.9. Solubility Parameter

The Hildebrand solubility parameter (*δ*) of a material is defined as the square root of the cohesive energy density as described by Equation (3):

(3) $$\delta =\;\sqrt{\frac{\Delta E_{V}}{V_{m}}}$$

where Δ*E_v_* represents the energy of vaporization and *V_m_* is the molar volume of the material [18]. When the solubility parameter cannot be determined experimentally, different methods may be used for estimating the *δ* value.

Estimated solubility parameters of CBD, PEG 400, virgin olive oil, and tributyl citrate were calculated using the method proposed by Fedors [37] Equation (4):

(4) $$\delta =\;\sqrt{\frac{\sum^{\;}\Delta e_{i}}{\sum^{\;}\Delta v_{i}}}$$

where Δ*e_i_* and Δ*v_i_* are the additive atomic and group contributions to energy of vaporization and molar volume, respectively. The solubility parameter of virgin olive oil was approximated to be equal to the *δ* value of triolein (triglyceride derived from three units of oleic acid) which represents its main component (up to 85%, according to the European Pharmacopoeia). Experimental *δ* values of purified water and propylene glycol, as well as estimated *δ* values of other materials, were taken from the literature [26,27,28,29], as reported in Table 3.

As the solubility parameter is an additive property, *δ* values of tested placebo formulations (Table 1) were derived according to Equation (5):

(5) $$\delta \;=\;\sum^{\;}\delta_{i}\varphi_{i}$$

where *δ_i_* is the solubility parameter of each excipient and *φ_i_* is its volume fraction [38]. The square of the difference between solubility parameters of the cannabinoid compound and placebo formulations (Δ*δ*^2^) was correlated with permeation study results.

### 4.10. Data Analysis

Tests for significant differences among means were performed by the one-way ANOVA followed by Turkey–Kramer post-analysis (JMP^®^ v14, SAS Institute Inc, Cary, USA). Differences were considered significant at the *p* < 0.05 level.

## 5. Conclusions

Given the scarce literature, this article consists of the first systematic collection of data on skin permeation of CBD. Ex vivo human tissue permeation parameters were examined following administration of the cannabinoid compound using both simple formulations, such as solutions and semisolid compositions, and more complex systems, such as a patch.

Among tested vehicle solutions, the propylene glycol/water mixture (80/20, *v*/*v*) achieved the best performance both in terms of permeation rate and skin retention. The transformation of the solution into hydrophilic gel did not affect the performance in terms of permeation rate but led to a significant reduction of the amount of CBD retained in the SCE. However, the hydrophilic gel proved to be the most effective vehicle among the proposed formulations. Paraffin oil and lipophilic ointment also proved to be suitable vehicles for the administration of CBD, guaranteeing a significant permeation performance. Despite lower dosing and unfavorable thermodynamic conditions, the (trans)dermal patch provided a comparable performance in terms of skin retention.

As regards solutions, it was observed that the solubility parameter can be used to predict performance in terms of CBD retention, a good correlation was found between Δδ^2^ and Q_r,24_ values; a trend was also observed in the case of semisolid formulations. This evidence reconfirms the importance of the choice of the solvent (or, more generally, of the composition of the vehicle) and demonstrates its influence on the permeant’s thermodynamic activity, and therefore on the partitioning process from the vehicle into the SC.

## Figures and Tables

**Table 1:** Cannabidiol (CBD) solubility in tested solutions (S_1_–S_4_); solubility parameters of placebo vehicles (δ) and Δδ^2^ values; in vitro CBD skin permeation parameters (steady-state flux (J), cumulative permeated amount at 24 h (Q_p,24_), retained amount at 24 h (Q_r,24_) and Q_r,24_/J ratio of tested solutions (S_1_–S_4_), and vehicle formulations (F_1_–F_3_). Means ± SD (*n* ≥ 3).

Vehicle	Solubility (mg/mL)	δ (cal/cm^3^)	Δδ^2^ (cal^2^/cm^6^)	J (μg/h/cm^2^)	Q_p,24_ (μg/cm^2^)	Lag time (h)	Q_r,24_ (μg/cm^2^)	Q_r,24_/J
S_1_	19.65 ± 0.40	7.09	16.5	0.99 ± 0.14	22.07 ± 3.92	1.79 ± 1.29	15.15 ± 4.02	15.28
S_2_	>300 *	8.93	4.93	0.02 ± 0.01	0.31 ± 0.20	>7	6.45 ± 3.23	351.13
S_3_	16.23 ± 0.51	16.54	29.03	1.06 ± 0.34	23.05 ± 6.79	1.95 ± 0.79	37.81 ± 9.20	35.69
S_4_	45.16 ± 1.04	13.34	4.35	0.03 ± 0.00	0.61 ± 0.04	3.37 ± 0.88	6.14 ± 1.46	207.45
F_1_	-	7.61	12.56	0.34 ± 0.06	7.23 ± 1.24	2.95 ± 0.67	8.30 ± 1.96	24.34
F_2_	-	16.51	28.72	1.28 ± 0.33	26.13 ± 7.24	3.84 ± 0.67	19.00 ± 5.31	14.89
F_3_	-	10.37	0.62	0.04 ± 0.02	0.93 ± 0.28	3.88 ± 1.43	4.99 ± 1.64	113.6

* Indicative value (*n* = 1).

**Table 2:** Composition of solutions and vehicle formulations.

	Composition (%, *w*/*w*)						
	S_1_	S_2_	S_3_	S_4_	F_1_	F_2_	F_3_
CBD	1.0	1.0	1.0	1.0	1.0	1.0	1.0
Liquid paraffin	99.0	-	-	-	49.0	-	-
Virgin olive oil	-	99.0	-	-	-	-	-
Propylene glycol	-	-	79.8	-	-	79.0	-
Purified water	-	-	19.2	17.9	-	18.0	-
PEG 400	-	-	-	81.1	-	-	-
White petrolatum	-	-	-	-	31.0	-	-
Cetostearyl alcohol	-	-	-	-	15.0	-	-
Cetomacrogol 1000	-	-	-	-	4.0	-	-
Hydroxyethyl cellulose	-	-	-	-	-	2.0	-
Eudragit RL	-	-	-	-	-	-	59.4
Tributyl citrate	-	-	-	-	-	-	39.6

**Table 3:** Solubility parameters (δ) of the components of the solutions and vehicle formulations.

Material	δ (cal/cm^3^)	Method of Calculation
CBD	11.15	Fedors
*O*-acylglucosylceramide	10.03 ^a^	Fedors
Liquid paraffin	7.09 ^a^	Fedors
White petrolatum	7.33 ^a^	Fedors
Triolein	8.93	Fedors
Cetomacrogol 1000	9.40 ^a^	Fedors
Cetostearyl alcohol	9.49 ^a^	Fedors
Tributyl citrate	10.35	Fedors
Eudragit RL	10.38 ^b^	Fedors
PEG 400	10.67	Fedors
Hydroxyethyl cellulose	12.25 ^c^	Fedors
Propylene glycol	14.80 ^d^	experimental
Purified water	23.50 ^d^	experimental

^a^ Minghetti et al. [26]; ^b^ Cilurzo et al. [27]; ^c^ Senta-Loys et al. [28]; ^d^ Burke [29].
